# The laser combined with intravitreal injection of ranibizumab for treatment of macular edema secondary to branch retinal vein occlusion: A protocol for systematic review and meta-analysis

**DOI:** 10.1097/MD.0000000000023675

**Published:** 2021-01-29

**Authors:** Guang Chen, Peng Chen, Xiaoping Chen, Jing Wang, Xinming Peng

**Affiliations:** Affiliated Hospital of Hebei University, Baoding, Hebei Province, China.

**Keywords:** branch retinal vein occlusion, laser, macular edema, ranibizumab, system evaluation

## Abstract

**Background:**

At present, laser is regarded as an effective treatment for macular edema secondary to branch retinal vein occlusion. With the breakthrough of anti-vascular endothelial growth factor drugs in ophthalmology clinical research, the intravitreal injection of ranibizumab is widely applied, but both methods have their limitations, so some clinical studies have combined and applied them together. However, the clinical results are inconsistent and controversial, and there is no relevant system evaluation for the laser combined with intravitreal injection of ranibizumab for treatment of macular edema secondary to branch retinal vein occlusion now.

**Objective:**

Meta analysis is used to analyze and evaluate the effectiveness and safety of the laser combined with intravitreal injection of ranibizumab for treatment of macular edema secondary to branch retinal vein occlusion.

**Method:**

CNKI, VIP, WANFANG, China Biology Medicine disc, Web of Science, PubMed, Embase, Cochrane Library have used random controlled clinical trial of laser combined with intravitreal injection of ranibizumab for treatment of macular edema secondary to branch retinal vein occlusion from the establishment of the database to October 2020. Two researchers conducted independent screening, quality assessment and data extraction for the literatures, and used RevMan5.3 to conduct Meta analysis for the included literatures.

**Result:**

The research has evaluated the effectiveness and safety of the laser combined with intravitreal injection of ranibizumab for treatment of macular edema secondary to branch retinal vein occlusion through the aspects of the best corrected visual acuity 6 months after operation, macular center thickness and the incidence of adverse reactions such as elevated intraocular pressure, endophthalmitis, vitreous hemorrhage and cataract.

**Conclusion:**

Laser combined with intravitreal injection of ranibizumab for treatment of macular edema secondary to branch retinal vein occlusion has good effect, and the research has provided reliable evidence for the use of clinical treatment of the laser combined with intravitreal injection of ranibizumab for treatment of macular edema secondary to branch retinal vein occlusion.

## 1 Introduction

Retinal vein occlusion is the second major disease in retinal vascular diseases, and branch retinal vein occlusion (BRVO) occupies about 80% of them, caused by arteriovenous cross compression, coagulation dysfunction and so on.^[1,2]^ BRVO can cause macular blood – impaired retinal barrier, so as to cause macular edema (ME). The macular area photoreceptor cell apoptosis caused by long-term ME is the common reason for the decrease of visual acuity of BRVO patients, and If it is not treated in time, it may cause irreversible damage on vision.^[3,4]^ Relevant research shows that venous obstruction will cause the obvious increase of the concentration of vascular endothelial growth factor, which is positively correlated with the severity of ME.^[5]^ Relevant clinical studies confirms that the anti-vascular endothelial growth factor (VEGF) drugs, such as laser, ranibizumab and so on, are the effective treatment of reducing ME and improving vision.

Laser therapy can improve retinal oxygen supply situation, relieve ME, and reduce vascular leakage, and at the same time, it can also accelerate the absorption of bleeding and promote the regression of edema subsided through throttling and diversion.^[8]^ VEGF drugs, such as ranibizumab and so on, promote the absorption of edema through inhibiting angiogenesis and reducing vascular exudation.^[9]^ However, both methods have their limitations, and laser photocoagulation of retina is easy to cause iatrogenic injury of retina. Anti-VEGF drugs need repeated injection to achieve stable effect, which have the risk of infection and related injection complications. Some clinical studies have combined the two, but the clinical results are inconsistent and controversial. Therefore, the study objectively has evaluated the effectiveness and safety of the laser combined with intravitreal injection of ranibizumab for treatment of ME secondary to BRVO, which has provided the scientific reference of the clinical application of the laser combined with intravitreal injection of ranibizumab for treatment of ME secondary to BRVO.

## 2 Methods

### 2.1 Protocol register

This protocol of systematic review and meta-analysis has been drafted under the guidance of the preferred reporting items for systematic reviews and meta-analyses (PRISMA). In addition, it has been registered on open science framework (OSF) on November 1, 2020 (Registration number: Doi: 10.17605/OSF.IO/HP4S6).

### 2.2 Ethics

There was no need to recruit patients and collect patient information, so ethics committee approval is not required.

### 2.3 Eligibility criteria

#### 2.3.1 Types of studies

We comprehensively collect the random controlled trial of laser combined with intravitreal injection of ranibizumab for treatment of ME secondary to BRVO. Regardless of region and publication situation, the language is limited to Chinese and English.

#### 2.3.2 Research object

The patients with macular edema secondary to branch retinal vein occlusion are definitely diagnosed through fundus angiography and optical coherence tomography, and there are no restrictions on nationality, gender and age.

#### 2.3.3 Interventions

The treatment group was treated with laser combined with intravitreal injection of ranibizumab; The control group was treated with other western medicine.

#### 2.3.4 Outcome indexes

(1) Primary outcome: the best corrected visual acuity 6 months after operation;
(2) Secondary outcomes: ① macular center thickness; ② the incidence of adverse reactions such as elevated intraocular pressure, endophthalmitis, vitreous hemorrhage and cataract.

### 2.4 Exclusion criteria

(1) Repetitively published literatures;
(2) Full-text papers unable to be obtained;
(3) Data with obvious errors or incomplete research;
(4) Random method or allocation of hidden bias risk is assessed as high-risk literatures.

### 2.5 Retrieval strategy

“Branch retinal vein occlusion”, “BRVO”, “ laser”, “ ranibizumab”, “macular edema”,“ME”are taken as the key words to conduct the retrieval in the Chinese database and English database including CNKI, VIP, WANFANG, China Biomedical Database, Web of Science, PubMed, Embase, Cochrane Library, and so on, which use random controlled clinical trial of laser combined with intravitreal injection of ranibizumab for treatment of macular edema secondary to branch retinal vein occlusion from the establishment of the database to October 2020. Taking PubMed as an example, the retrieval strategy is shown in Table 1.

**Table 1:** Retrieval strategy of PubMed.

Number	Search terms
#1	lasers[MeSH]
#2	laser[Title/Abstract]
#3	#1 OR #2
#4	ranibizumab[MeSH]
#5	ranibizumab[Title/Abstract]
#6	#4OR #5 OR #6
#7	retinal vein occlusion[MeSH]
#8	retinal vein occlusion[Title/Abstract]
#9	branch retinal vein occlusion[Title/Abstract]
#10	BRVO[Title/Abstract]
#11	#7 OR #8 OR #9 OR #10
#12	macular edema[MeSH]
#13	macular edema[Title/Abstract]
#14	ME[Title/Abstract]
#15	#12 OR #13 OR #14
#16	#3 AND #6 AND #11 AND #15

### 2.6 Data screening and extraction

**Figure 1:**
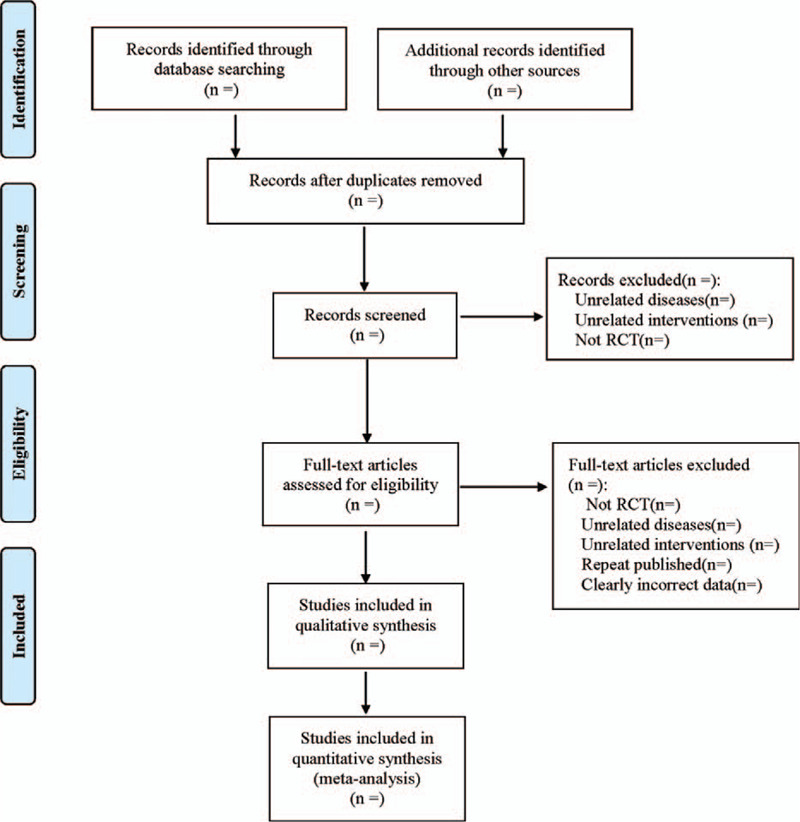
The process of literature screening.

Cochrane system evaluation manual is referred to be included into exclusion criteria according to PRISMA flowchart. It conducts independent screening for the retrieved literatures through EndNote X7 software by two researchers, and when there is a dispute on the inclusion of literatures, it should be negotiated or decided by a third researcher. Advance design information extraction table is used to extract the content of literatures, including ① Basic data: title, date of publication, author and literature source; ② Basic characteristics of research: the number of cases, intervention measures, follow-up, adverse events and so on; ③ Outcome indexes: the best corrected visual acuity 6 months after operation, macular center thickness, the incidence of adverse reactions such as elevated intraocular pressure, endophthalmitis, vitreous hemorrhage and cataract. The screening process is shown in Figure 1.

### 2.7 Literature quality evaluation

The inclusion study is based on the bias risk assessment tool in Cochrane 5.1.0 to conduct quality evaluation of methodology, and the aspects of Random sequence generation, Allocation concealment, Blinding of participants and personnel, Blinding of outcome assessment, Incomplete outcome Data, Selective reporting and Other bias are given judgment of risk degree and cross-check evaluation results by 2 researchers. If there are differences, they will be discussed and solved, and if there is no consensus has been reached, it should be discussed with the third researcher. Finally, Revman 5.3 is used to assess the risk of bias.

### 2.8 Statistical analysis

Revman 5.3 software in Cochrane collaboration network is used to conduct Meta analysis. The relative risk is used to conduct comparison of binary variables. If the measuring tool is consistent with the unit of measurement, the weighted mean difference should be used to represent a continuous variable. In case of inconsistency, the standard mean difference is adopted, and all effect amount are expressed by 95% confidence interval. Owing to the heterogeneity of the results evaluated by χ^2^ and *I*^*2*^ values, when *P *≥* *.1 and *I*^*2*^* *≤ 50%, it shows good homogeneity, adopting fixed effect model for analysis. If *P *<* *.1 and *I*^*2*^* *>* *50%, it shows that there is heterogeneity among the studies, and random effect model is used to analyze the sources of heterogeneity. Clinical heterogeneity should be treated through subgroup analysis, and if there is significant clinical heterogeneity and subgroup analysis was not available, Meta analysis shall not be conducted, and only descriptive analysis is conducted.

#### 2.8.1 Dealing with missing data

If there is data missing in the included literature, relevant test data can be achieved through contacting the researchers through email and other methods. If there is no contact has been made, or the researcher has lost the relevant data, Meta analysis shall not be conducted, and only descriptive analysis is conducted.

#### 2.8.2 Subgroup analysis

Subgroup analysis is conducted according to the treatment course, and subgroup is analyzed according to the treatment methods of the control group.

#### 2.8.3 Sensitivity analysis

In order to ensure the stability of outcome indexes, Sensitivity analysis of each outcome indexes should be conducted.

#### 2.8.4 Assessment of reporting biases

If the number of included literatures with the outcome indexes are more than or equal to 10, funnel plot was used to evaluate publication bias. In addition, Egger and Begg test were used for the evaluation of potential publication bias.

## 3 Discussion

BRVO is the most common retinal vascular disease in retinal vein occlusion, mainly expressing as retinal hemorrhage, edema and exudation, and If macular area is involved, it will cause serious vision loss.^[10,11]^ According to relevant reports, ME is the main reason of vision loss in patients with BRVO.^[12]^ Most of ME occurred in the early stage after occlusion from 1 month to several months. In the early stage of ME, with the disappearance of edema, the vision of most patients will have different degrees of recovery, and Severe or more than 8 months ME can cause photoreceptor ell apoptosis, so as to lead irreversible damage of cone cells. The visual acuity of the patient is seriously decreased and could not be restored, leading to permanent visual impairment. The study showed that the concentration of VEGF is positively correlated with the severity of ME.^[5,12–14]^ The duration of ME and the rate of edema absorption are very important for the prognosis of visual acuity. Long-term ME may also cause complications such as macular epiretinal membrane or macular hole.^[15]^ Therefore, it can effectively control the absorption of ME, reduce the concentration of VEGF, and promote the absorption of retinal hemorrhage infiltration, which have important significance to improve vision.

At present, the treatment of ME includes laser photocoagulation, intravitreal injection of anti-VEGF drugs and so on. Laser photocoagulation is mainly achieved by reconstructing the balance of retinal oxygen supply. The main principle is to destroy the photoreceptors of the hypoxic retina, increasing oxygen supply to undamaged sites and reducing capillary permeability, so as to reduce VEGF concentration and neovascularization. At the same time, laser photocoagulation can cause local retinal adhesion and promote the permeation of blood oxygen into the inner layer of retina to reach the objective of promoting the absorption of edema, hemorrhage and exudation.^[8,16]^ In recent years, breakthrough has been achieved for the anti-VEGF drugs in ophthalmic clinical research, and related clinical studies have shown that ranibizumab is effective in the treatment of ME secondary to BRVO. Ranibizumab is a high affinity recombinant monoclonal antibody fragment, which has targeted inhibition for the combination of VEGF-A and VEGFR-1 and VEGFR-2, so as to inhibit the neovascularization, and reduce the vascular exudation, promote the absorption of edema and achieve the purpose of treatment.^[17,18]^

Both laser photocoagulation and intravitreal injection of ranibizumab have their limitations, and laser is easy to cause iatrogenic retinal damage. The study have shown that anti-VEGF drugs need repeated injections to achieve stable effect, and at the same time, there is a risk of infection and related injection complications.^[19]^ Some clinical studies have combined the two, and the clinical results are inconsistent and controversial. Therefore, it is necessary to conduct analysis for the research of existing laser combined with intravitreal injection of ranibizumab in the treatment of RCT secondary to BRVO, so as to objectively evaluate the clinical efficacy and safety of laser combined with intravitreal injection of ranibizumab. Due to the number of included studies and the quality of literature, this systematic review still has its limitations, and at the same time, it is limited by language ability. We only search Chinese and English literature, ignoring the research in other languages. Therefore, more large sample and high-quality random double-blind controlled trials are needed to further confirm the effectiveness and safety of laser combined with intravitreal injection of ranibizumab.

## Author contributions

**Data curation:** Guang Chen, Peng Chen.

**Funding acquisition:** Xinming Peng.

**Software:** Xiaoping Chen.

**Supervision:** Jing Wang.

**Writing – original draft:** Guang Chen, Peng Chen.

**Writing – review & editing:** Xinming Peng.

## Abbreviations

Abbreviations: BRVO = branch retinal vein occlusion, CI = confidence interval, CNKI = China National Knowledge Infrastructure, GRADE = the grading of recommendations assessment, development, and evaluation, ME = Macular edema, OSF = open science framework, PRISMA-P = the preferred reporting items for systematic reviews and meta-analyses protocols, RCTs = randomized controlled trails, RR = relative risk, SMD = standardized mean difference, VEGF = vascular endothelial growth factor, VIP = China Science and Technology Journal Database, WMD = weighted mean difference.
